# Simultaneously Inhibiting BCL2 and MCL1 Is a Therapeutic Option for Patients with Advanced Melanoma

**DOI:** 10.3390/cancers12082182

**Published:** 2020-08-05

**Authors:** Nabanita Mukherjee, Carol M. Amato, Jenette Skees, Kaleb J. Todd, Karoline A. Lambert, William A. Robinson, Robert Van Gulick, Ryan M. Weight, Chiara R. Dart, Richard P. Tobin, Martin D. McCarter, Mayumi Fujita, David A. Norris, Yiqun G. Shellman

**Affiliations:** 1 Department of Dermatology, School of Medicine, University of Colorado Anschutz Medical Campus, Mail Stop 8127, Aurora, CO 80045, USA; Nabanita.Mukherjee@cuanschutz.edu (N.M.); jenette.skees@cuanschutz.edu (J.S.); kjtodd@fortlewis.edu (K.J.T.); karoline.lambert@cuanschutz.edu (K.A.L.); mayumi.fujita@cuanschutz.edu (M.F.); david.norris@cuanschutz.edu (D.A.N.); 2 Division of Medical Oncology, School of Medicine, University of Colorado Anschutz Medical Campus, Mail Stop 8117, Aurora, CO 80045, USA; carol.amato@cuanschutz.edu (C.M.A.); william.robinson@cuanschutz.edu (W.A.R.); robert.vangulick@cuanschutz.edu (R.V.G.); ryan.weight@cuanschutz.edu (R.M.W.); chiara.dart@colorado.edu (C.R.D.); 3 Division of Surgical Oncology, Department of Surgery, University of Colorado Anschutz Medical Campus, Aurora, CO 80045, USA; richard.tobin@cuanschutz.edu (R.P.T.); martin.mccarter@cuanschutz.edu (M.D.M.); 4 Dermatology Section, Department of Veterans Affairs Medical Center, Denver, CO 80220, USA; 5 Gates Center for Regenerative Medicine, University of Colorado Anschutz Medical Campus, Aurora, CO 80045, USA

**Keywords:** melanoma, BRAF-mutant, BRAF-WT, BH3 mimetics, venetoclax

## Abstract

There is an urgent need to develop treatments for patients with melanoma who are refractory to or ineligible for immune checkpoint blockade, including patients who lack BRAF-V600E/K mutations. This is often the case in patients diagnosed with rare melanoma subtypes such as mucosal and acral melanoma. Here, we analyzed data from the cutaneous melanoma The Cancer Genome Atlas Network (TCGA) transcriptomic and proteomic databases for differential expression of apoptosis molecules between melanomas with or without BRAF hotspot mutations. Our data indicated higher B-cell CLL/lymphoma 2 (BCL2) expression in melanoma without BRAF hotspot mutations, suggesting that BH3 mimetics, such as ABT-199 (venetoclax, a small molecule against BCL2), may be a potential therapeutic option for these patients. We explored the efficacy of combining two BH3 mimetics, ABT-199 and a myeloid cell leukemia sequence 1 (MCL1) inhibitor (S63845 or S64315/MIK665) in cutaneous, mucosal and acral melanomas, in vitro and in vivo. Our data indicate this combination induced cell death in a broad range of melanoma cell lines, including melanoma initiating cell populations, and was more potent in melanoma cells without BRAF-V600E/K mutations. Our knockdown/knockout experiments suggest that several pro-apoptotic BCL2 family members, BCL2-like 11 (apoptosis facilitator) (BIM), phorbol-12-myristate-13-acetate-induced protein 1 (NOXA) or BID, play a role in the combination-induced effects. Overall, our study supports the rationale for combining an MCL1 inhibitor with a BCL2 inhibitor as a therapeutic option in patients with advanced melanoma.

## 1. Introduction

BH3 mimetics are a novel class of anti-cancer drugs that mimic the action of certain BH3-only anti-apoptotic proteins of the B-cell CLL/lymphoma 2 (BCL2) family, which directly initiate apoptosis, downstream of many common oncogenes [1,2,3,4]. In the last five years, the BH3 mimetic, ABT-199 (venetoclax, a potent small molecule inhibitor against BCL2) has proven to be highly effective for treating advanced hematological malignancies [5]. However, single agent BH3 mimetics have shown inconsistent efficacy in solid tumors [6,7]. Currently, ABT-199 is the only FDA-approved BH3 mimetic. Clinical trials using ABT-199 plus another anti-apoptotic member of the BCL2 family, a BH3 mimetic targeting myeloid cell leukemia sequence 1 (MCL1), are underway to extend the efficacy of ABT-199 in hematological malignancies. The use of these compounds in solid tumors is being investigated in the pre-clinical phase [5,8,9].

While the efficacy of signal transduction inhibitors that target BRAF-V600E/K mutation is well described for patients with melanoma, treatments for patients that do not harbor a BRAF-V600E/K mutation are limited to immunotherapy and lack other clinical options [10]. Multiple studies have indicated the therapeutic potential of BH3 mimetics for cutaneous melanomas, including a seminal paper on the genomic landscape published by The Cancer Genome Atlas Network (TCGA) in 2015 [11]. However, the TCGA consortium did not explore treatment efficacy using in vitro or in vivo experiments.

In this study, we analyzed the cutaneous melanoma TCGA transcriptomic and proteomic database for differential expression of apoptosis molecules between melanomas with or without BRAF hotspot mutations. We refer to the genotypes as “BRAF-mutated (MUT)” or “BRAF-wild-type (WT)” for simplicity. Our data show that BCL2 is a potential therapeutic target for patients with the BRAF-WT genotype. We then explored the efficacy of combining two BH3 mimetics, an MCL1 inhibitor (S63845 or S64315/MIK665) and a BCL2 inhibitor (ABT-199/venetoclax) in cutaneous, mucosal and acral melanomas in vitro and in vivo. Overall, our study supports the rationale for combining clinic-ready BH3 mimetics as a therapeutic option in patients with advanced melanoma.

## 2. Results

### 2.1. TCGA mRNA and Protein Expression Analyses Suggest BCL2 as a Potential Therapeutic Target for BRAF-WT Melanomas

**Figure 1:**
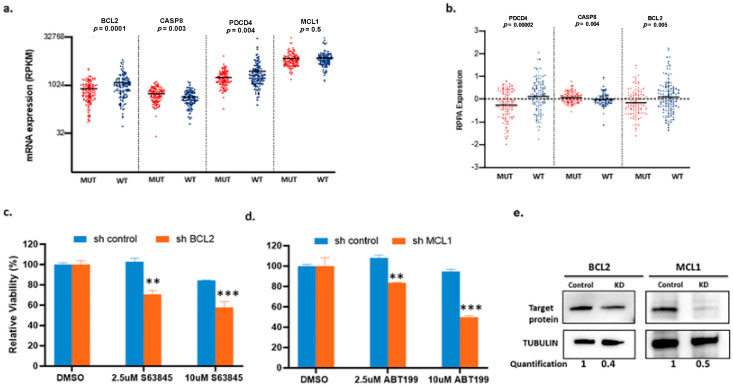
Differential expression of apoptotic-related mRNA and proteins in The Cancer Genome Atlas Network (TCGA) cutaneous melanoma dataset and the effects of knocking down B-cell CLL/lymphoma 2 (BCL2) or myeloid cell leukemia sequence 1 (MCL1). (**a** and **b**) Comparison between two genotypic groups: BRAF-hotspot mutated (MUT) (*n* = 110) and BRAF-wild-type (WT) (harboring RAS hotspot mutated, any NF1 mutated, and triple wild type *n* = 122). (**a**) mRNA expression values for BCL2, CASP8, PDCD4, and MCL1. (**b**) Relative reverse phase protein array (RPPA) protein expression values for PDCD4, CASP8, and BCL2. MCL1 was not included on the RPPA panel. Each dot represents an individual sample, and the horizontal line represents the mean. (**c**) and (**d**) show the effects of BCL2 or MCL1 knockdown in A375 cells. Cells were treated with the indicated drugs for 48 h. Knocking down BCL2 (shBCL2) sensitized cells to MCL1 inhibitor S63845 and knocking down MCL1 (shMCL1) sensitized cells to BCL2 inhibitor ABT-199. Y-axis shows percentage of relative viability and X-axis indicates the BH3 mimetics used. ** indicates *p* < 0.01; *** indicates *p* < 0.001. Error bars represent +/− SEM. (**e**) Immunoblots to confirm the knockdown.

Patients with BRAF-WT melanomas largely lack molecularly targeted treatment options. To identify other potential therapeutic targets, we analyzed the TCGA data for differences in gene and/or protein expression between BRAF-MUT and BRAF-WT melanoma. We utilized samples from the cutaneous melanoma dataset. Patient groups were defined and classified based on the original parameters set forth in the original TCGA paper [11], and we included samples that had both whole transcriptome (RNA-seq) and reverse phase protein array (RPPA) data available. Using these criteria, we identified 110 samples with a BRAF hotspot mutation and 122 samples with BRAF-WT for further analyses. We took a targeted approach in analyzing the RNA-seq data and focused on over 200 genes involved in the apoptosis pathway (Table S1). The RPPA panel in TCGA was limited and did not include MCL1 (Table S2). The only concordant results between these two analyses were higher levels of BCL2 and PDCD4, with a lower level of CASP8 in the WT group, compared with the MUT group (Figure 1a,b). Considering that higher BCL2 levels are associated with sensitivity to ABT-199 in other cancers [12,13,14], these data provide a rationale for testing the efficacy of BCL2 inhibitors, such as ABT-199 (venetoclax), in patients with BRAF-WT melanomas.

### 2.2. The Combination of the BCL2 Inhibitor ABT-199 with the MCL1 Inhibitors S63845 Has High Efficacy in BRAF-WT Melanomas In Vitro

Previously published work has shown that single agent BH3 mimetics are not effective alone for melanoma, and that MCL1 is an essential anti-apoptotic protein [6,7]. The combination of MCL1 inhibition with ABT-199 displayed efficacy in neuroblastoma with high BCL2 expression in vitro and in vivo [15]. In melanoma, knocking down BCL2 sensitized cells to the MCL1 inhibitor S63845, and conversely knocking down MCL1 sensitized cells to the BCL2 inhibitor ABT-199 (Figure 1c–e). Thus, these results suggest that the simultaneous targeting of both BCL2 and MCL1 is an effective combination to kill melanoma.

**Figure 2:**
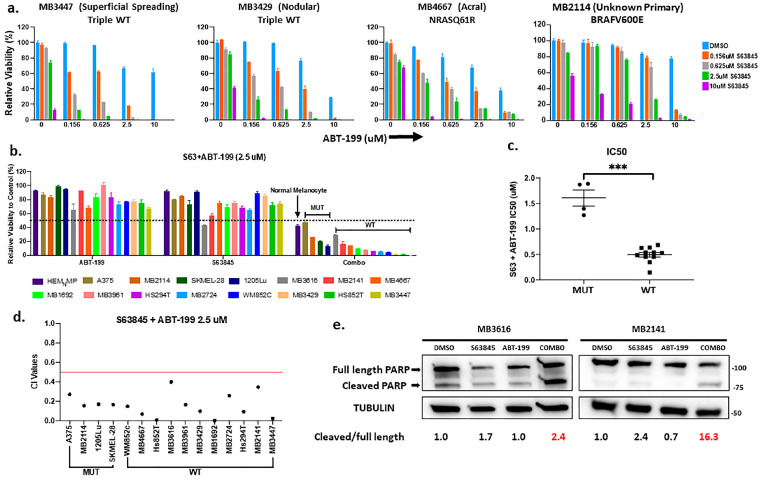
The combination of the BCL2 inhibitor ABT-199 with the MCL1 inhibitor S63845 had high efficacy in advanced melanomas in vitro. (**a**) ATP assays for various subtypes of melanoma patient samples and normal human melanocytes upon indicated treatments for 48 h. The viability of the DMSO control for each cell line was set to 100%. The viability of almost all combinational treatments was statistically significantly different from those of control or single drugs. For clarity and readability, we listed *p* values of all comparisons in Table S4 instead of showing an asterisk on figure. (**b**) Summary of ATP assay data of fifteen melanoma cell lines and patient samples and one primary melanocyte line treated with vehicle, single drug or combination of S63845 + ABT-199 at a dose of 2.5 μM. Black dotted line indicates 50% relative viability. (**c**) Dot plot of IC50 values for combination treatment in BRAF-V600E-MUT and BRAF-WT lines. Each dot represents one cell line. (**d**) Dot plot with the Combination-Index (CI) of the drug combination at 2.5 μM dose calculated using Compusyn software (version 1) (http://www.combosyn.com/) CI values < 0.5 (red line) indicate very strong synergism. Smaller CI values indicate stronger synergy. For visual clarity, the * is not shown in panels a and b. (**e**) Immunoblot with lysates collected after 48 h treatment with DMSO, single drugs, or combination at 0.625 μM for each drug, and probed for poly ADP-ribose polymerase (PARP). The ratio of cleaved/full-length PARP in DMSO control was set as 1 within each cell line, and the relative ratios in all conditions were normalized to DMSO controls and listed at the bottom. The combination increased the ratio of cleaved/full-length PARP in both cell lines, indicating that apoptosis was activated. Molecular weight markers are in kDa. *** indicates *p* < 0.01.

We tested the treatment efficacy of combining MCL1 inhibitors with ABT-199 in melanomas with or without BRAF-V600 hotspot mutations (MUT vs WT groups). A panel of patient-derived cell lines was also tested, and these include genetically diverse samples from patients with rare melanoma subtypes (mucosal and acral), and from patients who relapsed from various therapies (Table S3). We first utilized ATP assays to examine the in vitro viability following the treatments with S63845 and ABT-199, either as a single agent or in combination, in a panel of fifteen human melanoma lines and primary melanocytes (Figure 2a–d). Figure 2a shows a panel of melanomas treated with increasing concentrations of each BH3 mimetic by itself or in combination. Overall, single drug treatments of either ABT-199 or S63845 alone (up to 2.5 μM), had little effect on cell viability. Conversely, we saw a reduction in relative viability with combination therapy (Figure 2a–d and Table S4). Additionally, there was minimal effect on human primary melanocytes (Figure 2b). Interestingly, the combination treatment showed a greater efficacy on the BRAF-WT melanomas, as compared to the melanomas with BRAF-V600E (MUT). This similar trend was observed for the combination at a low dose, such as 0.625 μM (Figure S1). The mean half maximal inhibitory concentration IC50 of the combination was 0.5 μM for BRAF-WT, and the mean IC50 was 1.6 μM, more than 3-fold the IC50 for BRAF-MUT melanomas (Figure 2c and Table S3). Our analyses demonstrate the synergistic effects of this combination, calculated as a combination-index value (Figure 2d and Figure S1b). The combination also increased the ratio of cleaved/full-length poly ADP-ribose polymerase (PARP) and caused rounded morphology of cells (Figure 2e, Figure S2), indicating the induction of apoptosis. Taken together, these data indicate that this combination was effective to kill a wide range of melanomas, however this is more potent in BRAF-WT samples than with BRAF-MUT samples.

### 2.3. The Combination of ABT-199 with S63845 Effectively Slowed Tumor Growth In Vivo

**Figure 3:**
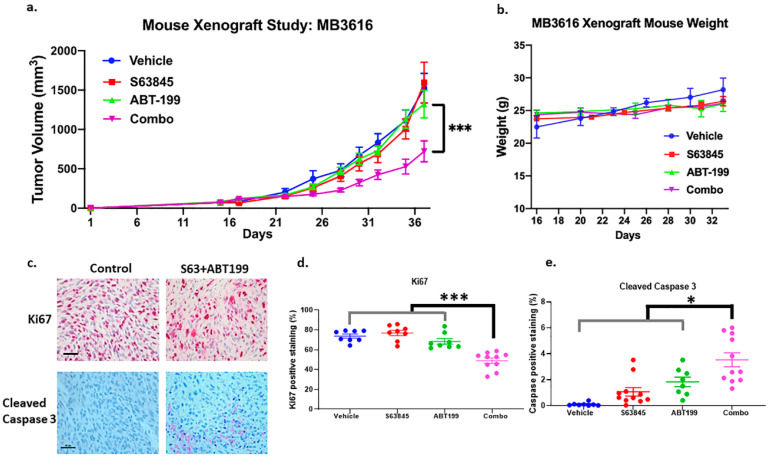
The treatment of ABT-199 plus S63845 significantly inhibited tumor growth without affecting mouse weight. A BRAF-WT line, MB3616, was used in the xenograft study, and tumor volume (**a**) and weight of the mice (**b**) were measured. The combination’s inhibiting effects on tumor volume was statistically significant, compared to vehicle or the single drugs (*p* < 0.001) (**a**). (**c**) Shows representative bright-field images of Ki67 and Cleaved Caspase 3 staining from tumor sections derived from mouse xenografts experiments. Scale bar, 50 μm. The summary quantifications are in (**d**) for Ki67 and (**e**) for cleaved Caspase 3 positive area. The effects of the combination were statically significant, compared to vehicle or individual treatments, and we only show the least significant p value of the comparisons. * indicates *p* < 0.05; *** indicates *p* < 0.001. Error bars represent +/− SEM.

We next evaluated the in vivo efficacy of the combination therapy in a mouse xenograft model of MB 3616, which has a NRAS-Q61K mutation and does not have a BRAF-V600E/K mutation (Figure 3a,b). The combination treatment significantly inhibited tumor growth when compared to the control or single drug treatments (*p* < 0.001) (Figure 3a). There were no significant changes in mouse weight (Figure 3b), suggesting no obvious adverse effects. Immunohistochemistry for cleaved Caspase 3 (an apoptosis marker) and Ki67 (a proliferation marker) on the tumor sections showed that the combination treatments significantly decreased Ki67 positive cells (*p* < 0.001) and increased the Cleaved Caspase 3 positive cells (*p* < 0.05) (Figure 3c–e, and Figure S3). Immunoblot of lysates from the tumors post-treatment showed that S63845 alone increased MCL1 expression 2.9-fold (Figure S4), which has been reported previously by others [16,17]. The combination treatment reduced this induction of MCL1 to only 1.5-fold (Figure S4). All of the treatments decreased BCL2 expression 40–60% (Figure S4).

Our in vitro data suggested that the combination of an MCL1 inhibitor and ABT-199 was effective against all melanomas, but higher concentrations were necessary for the BRAF-MUT compared to the BRAF-WT (Figure 2 and Figure S1). In vivo, this combination was also successful in inhibiting tumor growth for BRAF-V600E melanoma, when ABT-199 was administered at an increased frequency of three times per week (Figure S5a). There was minimal toxicity in our mouse xenograft model. Even with the higher frequency of dosing, there were no significant changes in mouse weight (Figure S5b) and the mice were healthy overall during regular monitoring throughout the duration of the studies. These results collectively indicate that the combination of the MCL1 inhibitors with the BCL2 inhibitor ABT-199 is effective in killing advanced melanomas.

### 2.4. The Combination of ABT-199 with S63845 Significantly Inhibited Sphere-Forming Capacity of the Melanoma Initiating Cells

In melanoma, a sub-population of cells has enhanced plasticity, drug resistance and stem-cell-like features. These cells are referred to as Melanoma Initiating Cells (MICs) and may contribute to drug resistance and relapse [18,19]. Although ABT-199 has been shown to kill leukemia stem cell populations [20,21], this effect has not been reported for MICs. We employed two commonly used methods, primary and secondary sphere forming assays [22,23,24,25,26,27]. The primary sphere assay enriches MIC populations, and can be used to measure the effects of drug treatments on MIC populations [26,27,28,29], whereas the secondary sphere assay measures the self-renewal capacity of the MICs after initial treatment [26,28].

**Figure 4:**
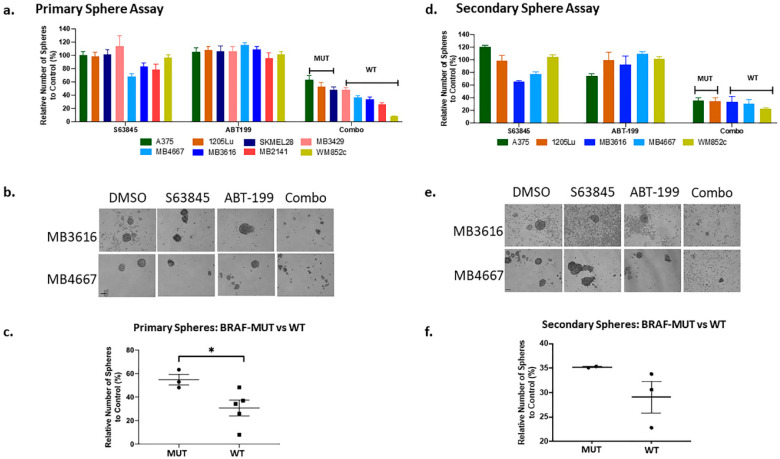
The combination of ABT-199 with S63845 significantly inhibited sphere-forming capacity of the Melanoma Initiating Cells. Melanoma cells were subjected to the primary sphere assay (**a**–**c**). Spheres were treated with the indicated compounds either alone, or in combination, for 48 h, and the number of surviving spheres were counted and quantified (**a**). (**b**) Shows example images by phase contrast microscopy. (**c**) Dot plot of normalized primary sphere (expressed as percentage) for combination treatment in BRAF-V600E MUT and WT lines. Secondary sphere assay was conducted with surviving cells from each treatment conditions from the primary sphere assay (**d**–**f**). (**d**) Quantification of the number of secondary spheres; (**e**) the images of a representative secondary sphere, and (**f**) dot plot of the relative number of secondary spheres in the combination wells for BRAF-V600E MUT and WT lines. In all melanoma lines, the combination treatment significantly reduced the primary and secondary sphere formation compared with all other treatments (DMSO or single drug). For visual clarity, we have not marked the significance in panel (**a**) and (**d**). * indicates *p* < 0.05. Error bars represent +/− SEM. For clarity and readability of panels a and b, we listed *p* values in Table S5. Scale bar = 100 μm.

The combination therapy significantly disrupted primary spheres (Figure 4a,b and Table S5). Similar to the ATP assay (Figure 2c), the combination was more successful in inhibiting the primary spheres in lines with BRAF-WT (WT) genotypes than those with BRAF-V600E (MUT) (Figure 4c and Table S5). The combination treatment also eliminated almost all sphere formation in the secondary sphere assays, compared to DMSO or single drug treatment (*p* < 0.001) in all cell lines tested (Figure 4d,e); results suggest the combination effectively killed MICs and inhibited self-renewal capacity. There was no significant difference in secondary sphere formation ability between the BRAF MUT vs WT lines (Figure 4f). These results suggest that the combination of ABT-199 and S63845 can play an important role in preventing relapse caused by MICs.

### 2.5. The Effects of ABT-199 + S63845 Is Partially Dependent on Pro-Apoptotic BCL2 Family Members NOXA, BIM, and BID

**Figure 5:**
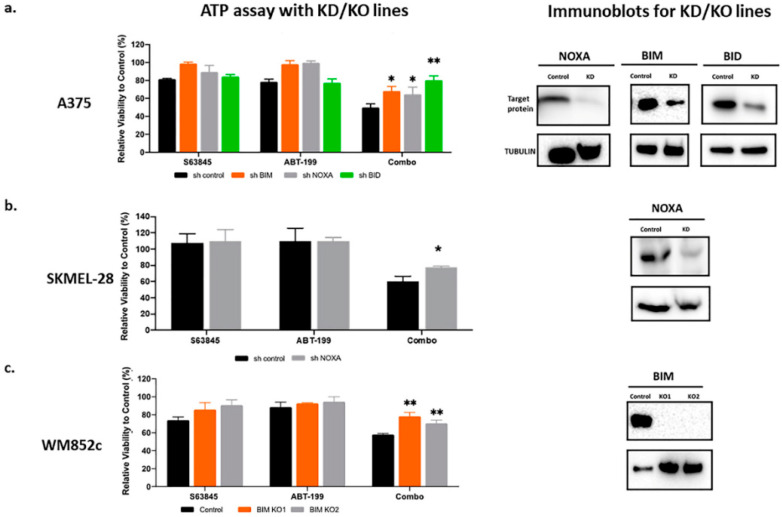
The combination-induced cell death was partially dependent on NOXA, BCL2-like 11 (apoptosis facilitator) (BIM) or BID. ATP assays with knockdown (KD) or knock out (KO) of indicated cells to test if the KD/KO protects against combination-induced cell death. Immunoblot to show the knockdown or knockout of NOXA, BIM or BID. (**a**) KD lines for NOXA, BIM, or BID in A375. (**b**) KD lines for NOXA in SKMEL-28. (**c**) BIM KO lines in WM852c. * indicates *p* < 0.05; ** indicates *p* < 0.01. Error bars represent +/− SEM.

The NOXA–BCL2-like 11 (apoptosis facilitator) (BIM)–MCL1 axis plays a crucial role in BH3 mimetic induced cell death [30]. In B cell lymphoma cells, genomic amplification or pharmacologic induction of NOXA sensitizes cells to BCL2 inhibitors, including ABT-199 [31]. In mantle cell lymphoma, both BIM and NOXA mediate ABT-199-induced cell death [32]. In acute myeloid leukemia (AML), BIM is an important mediator for S63845-induced apoptosis [33]. In melanoma, we and others have shown that NOXA and BIM mediate the killing effects of several combinations, including the BH3 mimetic ABT-737 and ABT-263 [6,34,35,36,37,38,39,40,41]. Thus, we investigated the roles of BIM and NOXA in the S63845+ABT-199 mediated cell death with cell lines genetically modified with shRNA or CRISPR-Cas9 technology. Knocking down or knocking out BIM or NOXA partially protected melanoma cells from the combination treatment but did not eliminate the killing effects (Figure 5a–c).

Another pro-apoptotic BCL2 family member, BID, is the only member that can be activated by CASP8, which is one of the genes identified in the TCGA analyses. Therefore, we performed knockdown experiments to investigate the role of BID in ABT-199 plus S63845 induced killing. Like NOXA and BIM, knockdown of BID also enhanced melanoma resistance to the combination (Figure 5a). Taken together, our data indicated that BIM, NOXA and BID all play some roles in the killing induced by this combination.

### 2.6. S64315 Has Similar Synergistic Effects as S63845, When Combined with ABT-199

**Figure 6:**
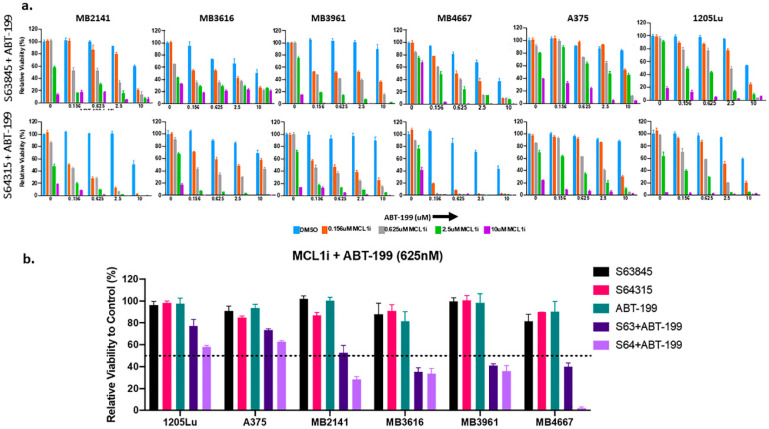
Combination therapy of S64315/MIK665 (the clinic-ready version of S63845) with ABT-199 has a synergistic effect in treating melanoma samples of diverse genetic backgrounds and is comparable to the effects of S63845 with ABT-199. (**a**) ATP assays of melanoma cell lines upon indicated treatments for 48 h. The viability of the DMSO control for each cell line was set to 100%. Both the combinations (S63845 + ABT-199; upper panel and S64315 + ABT-199, lower panel) had similar efficacy in reducing the cell viability of representative melanoma lines. (**b**) Summary of ATP assay data of six melanoma cell lines, including patient derived cell lines, treated with single drug or a combination of S63845 + ABT-199 or S64315 + ABT-199. All drugs were used at a dose of 625 nM. For visual clarity, the * is not shown in the figures. Both combinations were highly synergistic at sub-micromolar doses. Error bars represent +/− SEM. For clarity and readability, we listed *p* values in Table S6.

S64315 (MIK665), which is derived from S63845, has similar chemical properties to inhibit MCL1, and is currently in clinical trials for AML (ClinicalTrials.gov NCT02992483; NCT02979366; https://clinicaltrials.gov/ct2/show/NCT02992483; https://clinicaltrials.gov/ct2/show/NCT02979366). Thus, we performed a comparative analysis of S64315 and S63845, either alone or in combination with ABT-199 in multiple melanoma cell lines. These include both BRAF-WT and BRAF-MUT lines. Overall, S64315 exhibited similar or better efficacy than S63845 (Figure 6 and Table S6).

## 3. Discussion

Treatment options for patients with advanced cutaneous melanoma expanded significantly with FDA approval of immune-checkpoint blockade drugs and BRAF/MEK targeting signal transduction inhibitors. Patients with metastatic or unresectable melanoma treated with combination immune checkpoint blockade (Ipilimumab/Nivolumab) or BRAF/MEK inhibition demonstrate 5-year overall survival of 52% and 34%, respectively [42,43]. Despite these advances, a large proportion of patients treated with these medications will progress or not be eligible for targeted therapy. This includes those with BRAF-WT melanoma, and those who do not respond to immunotherapies. In addition, patients with rare melanoma subtypes, such as acral and mucosal, are genetically distinct from cutaneous melanomas and typically lack BRAF-V600E/K mutations, making them ineligible for BRAF/MEK inhibition [44]. A large proportion of these patients (30–60%) also do not respond to immunotherapies [45,46]. Therefore, there is a significant need to develop new treatments for these patients. Our study strongly suggests that combinations of BH3 mimetics that target both MCL1 and BCL2 may fill this role.

Our analyses of TCGA data identified higher expression of BCL2 in cutaneous BRAF-WT melanoma, compared to those with a BRAF hotspot mutation. These data led us to explore BH3 mimetics in advanced melanomas, especially those without BRAF-V600E/K. For this reason, we expanded our analysis to include rare melanoma subtypes of mucosal and acral, which often lack BRAF hotspot mutations. Our in vitro and in vivo data showed that the simultaneous targeting of BCL2 and MCL1 is effective in treating advanced melanoma, and this combination is more potent in melanomas without the BRAF-V600E/K variant. This observation is novel and provides a feasible approach for patients with limited therapeutic options.

We found that this combination was successful across our panel of melanoma cell lines. In addition, our in vitro data with viability assays and primary sphere assays show a trend of BRAF-WT melanomas being more sensitive that is, sensitive at a lower dose to this combination. For our BRAF-V600E melanoma in vivo model, we needed to utilize higher doses and/or increase the frequency of administration for these compounds to achieve similar results—i.e., a reduction in cell viability in vitro and inhibition of tumor growth in vivo (Figure 2, Figure 3 and Figures S1 and S5). There was minimal toxicity in vivo. Even with more frequent drug administration, we did not detect any obvious adverse health effects, such as weight loss, in our mouse xenograft model. These data indicate that the drugs should be delivered more frequently and/or at a higher concentration to achieve similar results in patients with BRAF mutated melanoma. However, this is possible only if this combination continues to show limited toxicity in human trials.

The combination of ABT-199 with MCL1 inhibitors has been studied in other cancers, both in vitro and in vivo [16,32,47,48,49,50,51], and is currently in clinical trials for patients with AML. However, the efficacy of this combination has never been studied thoroughly in melanoma. Lee et al., in 2019, reported the in vitro efficacy of the S63845 with ABT-199 in combination in a limited number of cutaneous melanoma cell lines [7]. We are the first to study the efficacy of the combination of S63845 with ABT-199 in vivo in melanoma. In addition, our sphere assay data suggest this combination may prevent relapse caused by MICs. Moreover, our current study also includes in vitro data that tests a clinic-ready version of the MCL1 inhibitor, S64315 (MIK665), in combination with the FDA approved ABT-199, further justifying the use of this therapeutic option for patients with advanced melanoma.

We and others have shown that navitoclax plus MCL1 inhibitors can be effective to kill melanomas in vitro and in vivo [6,7]; however, in our experience, the potential toxicity was higher than ABT-199 plus MCL1 inhibitors. In our previously published study, we had to lower the drug concentrations of navitoclax and MCL1 inhibitors to minimize toxicity [6]. In comparison, the combination of ABT-199 plus an MCL1 inhibitor has been tested in pre-clinical mouse studies and is considered safe even when administered at higher concentrations than what we demonstrate in this current study (S63845: 25 mg/kg twice weekly; ABT199: 50 mg/kg 2–3 times per week). For instance, Seiller et al. [52] used ABT-199 (100 mg/kg; 5 days/week) along with S63845 (25 mg/kg; every 6 days) for 19 days without significant weight loss in NOD scid gamma (NSG) mice. Prukova et al. [32] safely administered S63845 (25 mg/kg) and ABT-199 (50 mg/kg) simultaneously in a mouse model of AML for five days. Minimal toxicity was observed with other clinically approved MCL1 inhibitors (such as AZD5991), which were administered at higher doses and more frequently than in our current study [50,51]. In addition, ABT-199 is already FDA-approved for some cancers. Toxicity data for MCL1 and BCL2 inhibition in humans has not been reported to date, however, early phase clinical trials assessing safety are underway in non-melanoma tumor types. A phase 1 study (https://clinicaltrials.gov/ct2/show/NCT03672695) (NCT03672695) examining the combination of S64315 with ABT-199 is underway for patients with AML. The study protocol includes the weekly administration of S64315 (50 mg–1000 mg) and daily ABT-199 (100 mg–600 mg). Thus, our data along with current clinical trials provide the framework for testing the efficacy of the combination of MCL1 inhibitors with BCL2 inhibitors in melanoma patients.

BH3 mimetics promote cell death primarily through the intrinsic apoptotic pathway. Our data indicate that BIM, NOXA, and BID play a role in this combination treatment. BIM and NOXA’s roles are consistent with the primary apoptotic pathway promoted by BH3 mimetics [16,32]. Although knockdown of BID decreased cell sensitivity to this treatment, our data indicate that a BID-null state will not prevent killing completely, as we could not detect BID in the moderately sensitive cell line MB 2141 (Figure S6). The role of BID has only been reported in the effects induced by the BH3 mimetic ABT-737 [53,54,55]. BID provides a link between the extrinsic and intrinsic apoptotic pathways. These data indicate that crosstalk between these two apoptosis pathways is involved with BH3 mimetic treatments. Further studies are needed to better characterize this connection with the combination treatment of MCL1 inhibitor plus ABT-199.

To explore why BRAF-WT melanomas respond better to this combination, we examined the expression of various BCL2 family members. There was a higher expression of BIM in BRAF-WT melanoma, compared to BRAF-MUT melanoma, although it is not statistically significant (Data not shown; Unpublished work) [56]. This is consistent with previous finding that BRAF-V600E can downregulate BIM expression in melanoma [57,58]. Considering that our data showing BIM knockout or knockdown results in decreased melanoma sensitivity to this combination, we speculate that higher BIM expression may be a contributing factor for a better response in BRAF-WT melanoma. Future studies with more samples are needed to determine if this is true.

## 4. Materials and Methods

### 4.1. Analysis of the TCGA Cutaneous Melanoma Dataset

Data and clinical information were downloaded from the FireBrowse website (http://firebrowse.org/) and supplemental files provided in the TCGA cutaneous melanoma paper [11]. Multiple *t*-tests were performed using GraphPad v8 (https://www.graphpad.com/), discovery determined using the Two-stage linear step-up procedure of Benjamini, Krieger and Yekutieli, with *q* = 1%. Each gene or protein was analyzed individually, without assuming a consistent standard deviation.

### 4.2. Reagents and Drug Treatments

All drugs (S63845, S64315, and ABT-199) used for the study were purchased from MedChem Express (Monmouth Junction, NJ, USA) or from Selleck Chem (Houston, TX, USA). For the initial cell viability assays, each drug was tested at a dose range of 0.156 to 10 μM by itself or in combination, and then a dose of either 0.625 or 2.5 μM was used for the subsequent studies. Cells were treated for 48 h for viability assays and primary sphere assays.

### 4.3. Melanoma Cell Lines, Either Long-Established Conventional Lines or Newly Established Patient Lines

Patient derived cell lines were provided by the University of Colorado Skin Cancer Biorepository (patient consent and specimen usage outlined under COMIRB 05-0309) and validated by melanoma triple cocktail staining. Patient lines were derived from metastases of patients seen at our institution, and include samples derived from patients relapsed from current treatments. Patient lines were Short tandem repeat (STR) profiled with >80% match to the patient’s corresponding tumor or blood sample. Genetic backgrounds are listed in Table S3. All cell lines were maintained in RPMI 1640 medium (Invitrogen, Grand Island, NY, USA) with 10% fetal bovine serum (Gemini Bio-Products, Inc., West Sacramento, CA, USA) and were tested for mycoplasma. Primary melanocytes HEM_N_MP were obtained from Life Technologies (Carlsbad, CA, USA). Melanocytes were maintained in Medium 254 with Human Melanocyte Growth Supplement-2 (Life Technologies, Carlsbad, CA, USA). To mimic melanoma culture conditions, 10% FBS was added for drug assays.

### 4.4. ATP Viability Assay, Primary and Secondary Sphere Assays

Cell viability was evaluated via a Cell Titer-Glo Luminescent cell viability assay (Promega Corp., Madison, WI, USA) according to the manufacturer’s protocol. All sphere assays were completed as described in our previous publications [6,36,37,38,40]. The experimental schematic for the primary and secondary sphere assays was described previously [38]. All assays were performed in no less than triplicate for each cell line and repeated at least thrice for each line. Drug treatments began 120 h after seeding in the primary sphere assay and 24 h after seeding for the monolayer ATP assay.

### 4.5. Immunoblot

Both floating and adherent cells were collected and lysed using 2× Laemmli buffer (Bio-Rad, Hercules, CA, USA). Samples were analyzed using the immunoblot analysis protocol as described in [59,60]. Immunoblot images presented in this manuscript are from a representative experiment carried out in triplicate. The antibody dilutions were used at the manufactures’ recommendation. The following antibodies were purchased from Cell Signaling Technologies (Danvers, MA, USA): PARP (#9532), BID (#2002), BIM (#2933), BCL2 (#15071), α/β tubulin (#2148), and HRP-conjugated goat anti-mouse and anti-rabbit antibodies. The NOXA antibody (# OP180) was obtained from Millipore Sigma (St. Louis, MO, USA) and MCL1 antibody (#819) was purchased from Santa Cruz Biotechnology (Dallas, TX, USA). Please see Figures S7–S11 for uncropped images of immunoblots presented in the Main and Supplementary Figures.

### 4.6. Creation of Short Hairpin RNA Transduced Cell Lines and CRISPR/Cas9-Mediated BIM Knockout Cell Lines

ShRNA lentiviral particles (Santa Cruz Biotechnology, Dallas, TX, USA) were used to construct stable cell lines as previously described [60]. BIM-knockout lines were generated using CRISPR/Cas9 technology, as previously described [37].

### 4.7. Mouse Xenograft Studies

All animal experiments mentioned in this study were approved by the Institutional Animal Care and Use Committee (IACUC) of the University of Colorado Denver (protocol number 318). Similar mice and standard methodology were used for tumor implantation and tumor measurements, as in our previous studies [6]. Briefly, 6–8 weeks old NCRNU nude mice were used, with tumor cells injected subcutaneously in each flank with a 100 uL suspension of 2 to 3.5 million cells in 50% BD Matrigel (#354263, BD Biosciences, Billerica, MA, USA). Drug treatments started when the tumors were palpable. Treatment groups consisted of randomly divided mice of at least 8 tumors each group. All drugs were prepared according to the manufacturer’s protocol, or previously described [6]. S63845 was administered at 25 mg/kg twice weekly. ABT-199 was administrated 50mg/kg twice per week for the study with MB3616 cells and three times per week for the study of A375 cells. The tumor samples were collected at the end of the experiment for further studies. The doses used here were chosen based on a thorough literature search and our pilot experiments, and these doses were safe in similar doses for other cancers [32,50,51,52].

### 4.8. Immunohistochemistry (IHC)

The detailed procedure has been described in our previous publication [6]. The protocol was adapted from [61]. In short, the tumors were subjected to fixation and dehydration gradient before being embedded with paraffin. Sections of 4-μm thickness were used for staining in a Dako Autostainer described in [6]. The antibodies used in the study are Cleaved Caspase 3, (1:200, #9664, Cell Signaling Technology, (Danvers, MA, USA) and Ki67, (1:100, #RM-9106-S1, Thermo Fisher Scientific, (Waltham, MA, USA). The details of imaging and quantifications are also described in our previous publication [6].

### 4.9. Calculation of IC50 and Combination Index (CI) Values

IC50 values were calculated from the relative viability results of ATP assays, and were derived by a sigmoidal dose (log)-response (variable slope) curve with GraphPad Prism 8 software. The combination index was calculated to determine the synergistic effects of the combination treatment, using Compusyn software (version 1); values <0.9 indicate synergism and smaller CI values indicate stronger synergy [62].

### 4.10. Statistical Analysis

GraphPad Prism V8 software was used to make the graphs and the statistical analyses. We used *t*-tests or one-way ANOVAs followed by appropriate post-hoc tests to determine if the experimental groups were significantly different. Data of the mouse xenograft studies were analyzed by two-way/mixed model ANOVA followed by appropriate post-hoc tests.

## 5. Conclusions

In summary, our data indicate that targeting the apoptosis pathway using two different BH3 mimetics is effective in inducing cell death in melanomas. This combination of BH3 mimetics offers a potential therapeutic strategy for the treatment of advanced melanomas, including those without BRAF hotspot mutations. Additionally, our data suggests that this can be accomplished with minimal toxicity, however this needs further examination in the clinical setting. Therefore, this study can serve as the starting point for clinical trials that combines MCL1 inhibitors with BCL2 inhibitors for patients with advanced melanoma.

## Supplementary Materials

The following are available online at Supplementary Data https://www.mdpi.com/2072-6694/12/8/2182/s1, Figure S1: The combination of S63845 + ABT-199 kills BRAF-WT melanoma cell in vitro at sub-micromolar dose, Figure S2: S63845 combined with ABT-199 induced apoptosis in melanoma cells, Figure S3: Representative bright-field images of Ki67 and Cleaved Caspase 3 staining from tumor sections derived from mouse xenografts experiments of Figure 3, Figure S4: Immunoblot with tumor cell lysates collected from the mouse xenograft experiment of Figure 3, Figure S5: The combination of S63845 + ABT-199 kills BRAF-V600E melanoma cells in vivo at higher frequency of treatment, Figure S6: Endogenous level of BID in melanoma cell lines and patient samples, Figure S7: Full immunoblot images of bands shown in Figure 1e, Figure S8: Full immunoblot images of bands shown in Figure 2e, Figure S9: Full immunoblot images of bands shown in Supplementary Figure S4, Figure S10: Full immunoblot images of bands shown in Figure 5, Figure S11: Full immunoblot images of bands shown in Supplementary Figure S6, Table S1: Comparison of the mRNA expression from the TCGA cutaneous melanoma data set, Table S2: Comparison of the protein expression from the TCGA cutaneous melanoma RPPA data set, Table S3: Details of the melanoma lines used in the study and IC50 of indicated drugs, Table S4: *p* values for ATP assay of S63845+ABT-199 Combination (Figure 2a), Table S5: *p* values for Figure 4, Table S6: *p* values for Figure 6.

## Abbreviations

MCL1	Myeloid cell leukemia sequence 1
BCL2	B-cell CLL/lymphoma 2
BIM	BCL2-like 11 (apoptosis facilitator)
PARP	Poly ADP-ribose polymerase 1
NOXA	Phorbol-12-myristate-13-acetate-induced protein 1
BID	BH3 Interacting Domain Death Agonist
CASP8	Caspase 8
PDCD4	Programmed cell death protein 4
CRISPR	Clustered regularly interspaced short palindromic repeats
BRAF	B-Raf proto-oncogene
BH3	Bcl-2 Homology 3
ABT-199	Abbott Laboratories, a Bcl-2 selective BH3 mimetic
FDA	U.S. Food and Drug Administration
RAS	Rat sarcoma family of oncogenes
NF1	Neurofibromin 1
NRAS	Neuroblastoma RAS Proto Oncogene
SEM	Standard error of the mean
MEK	Mitogen-activated protein kinase family
