# Supplementary material for: Simultaneously Inhibiting BCL2 and MCL1 Is a Therapeutic Option for Patients with Advanced Melanoma
Source: Cancers (Basel). 2020 Aug 5;12(8):2182. doi: 10.3390/cancers12082182 (PMC7464298; doi:10.3390/cancers12082182)
Supplement: Supplementary file 1 [file cancers-12-02182-s001.zip › supplementary-author proofed-update/Supplemental Materials-2020-8-3-NM.docx]

Supplementary Figures


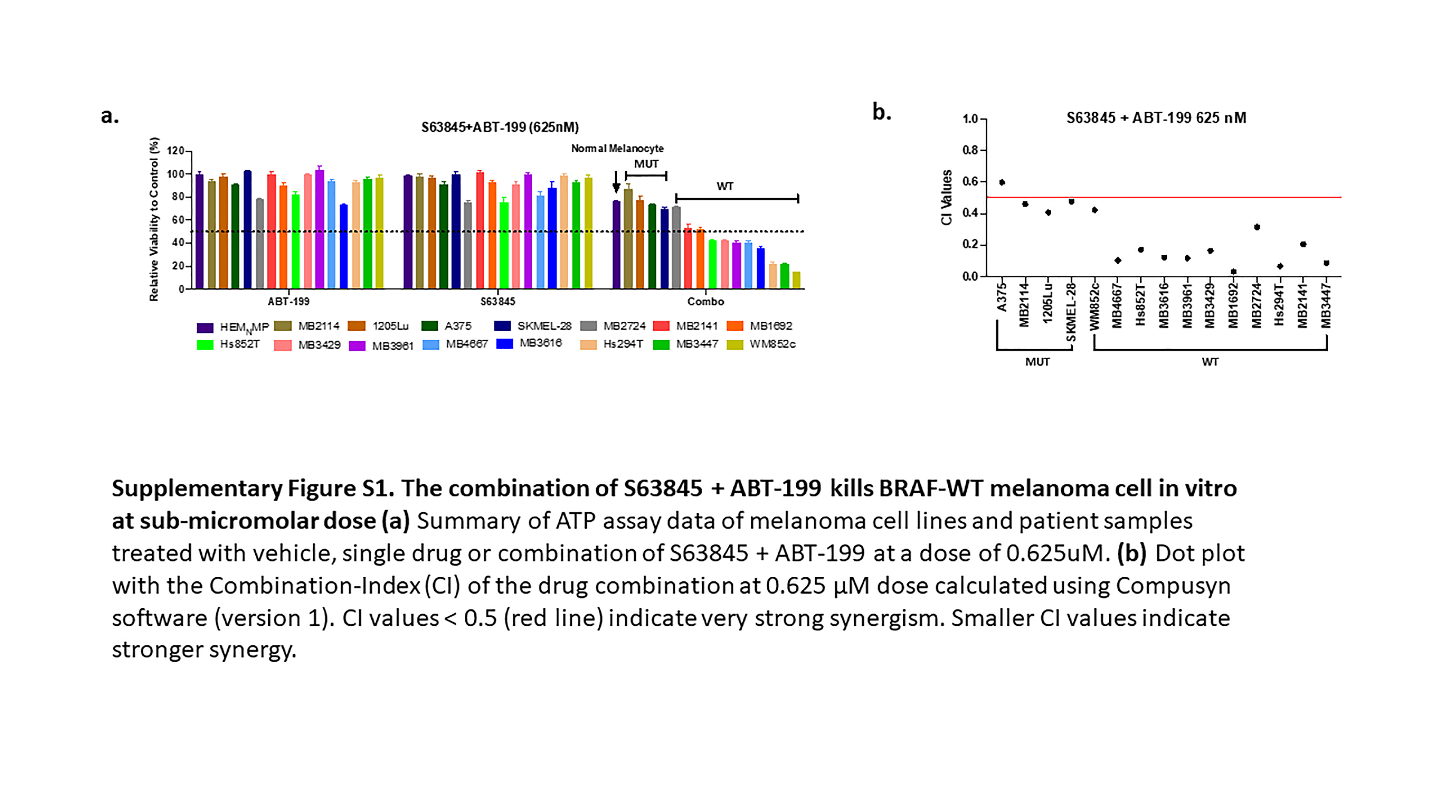


**Figure S1.** The combination of S63845 + ABT-199 kills BRAF-WT melanoma cell in vitro at sub-micromolar dose (**a**) Summary of ATP assay data of melanoma cell lines and patient samples treated with vehicle, single drug or combination of S63845 + ABT-199 at a dose of 0.625uM. (**b**) Dot plot with the Combination-Index (CI) of the drug combination at 0.625 μM dose calculated using Compusyn software (version 1). CI values < 0.5 (red line) indicate very strong synergism. Smaller CI values indicate stronger synergy.


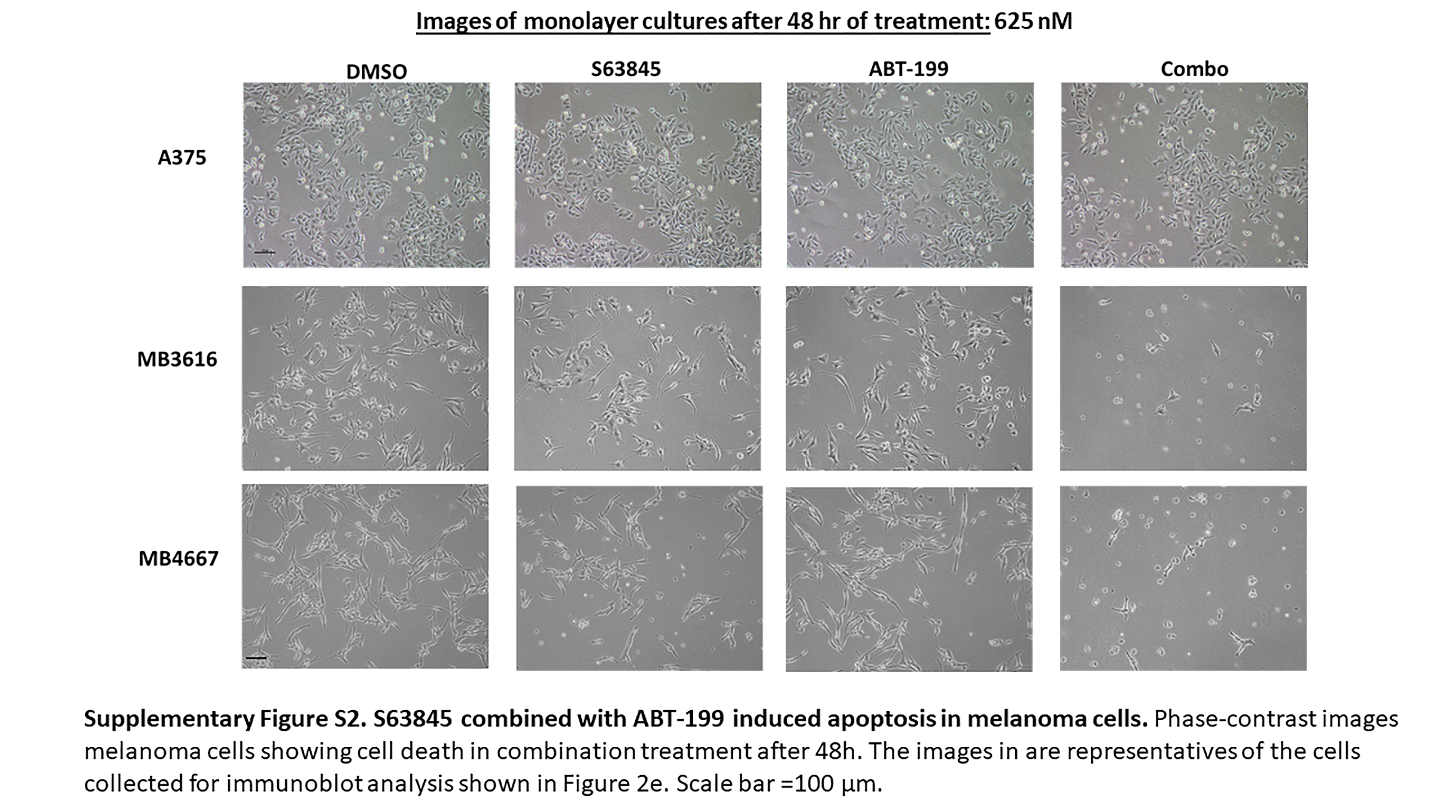


**Figure S2.** S63845 combined with ABT-199 induced apoptosis in melanoma cells. Phase-contrast images melanoma cells showing cell death in combination treatment after 48h. The images in are representatives of the cells collected for immunoblot analysis shown in Figure 2e. Scale bar = 100 μm.


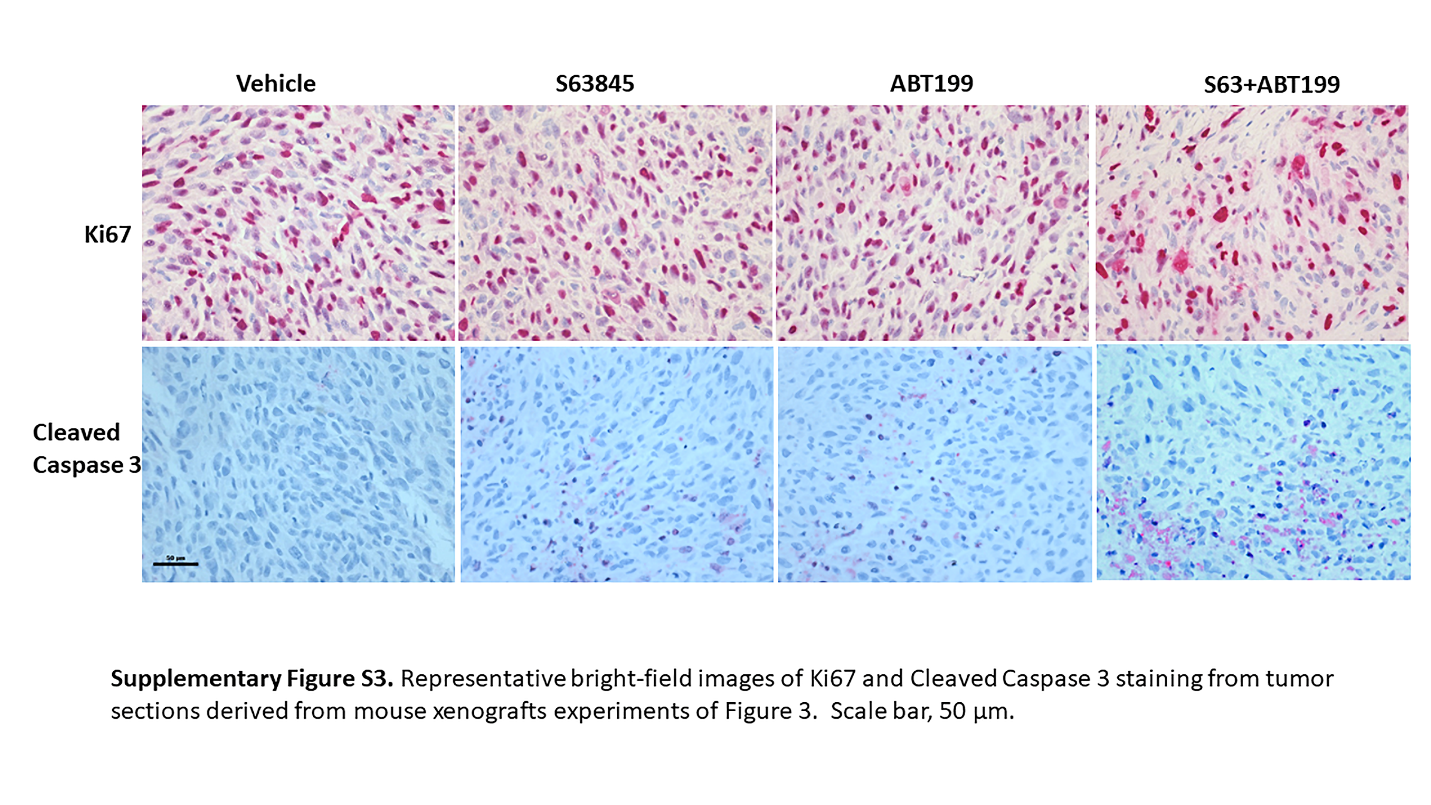


**Figure S3.** Representative bright-field images of Ki67 and Cleaved Caspase 3 staining from tumor sections derived from mouse xenografts experiments of Figure 3. Scale bar, 50 μm.


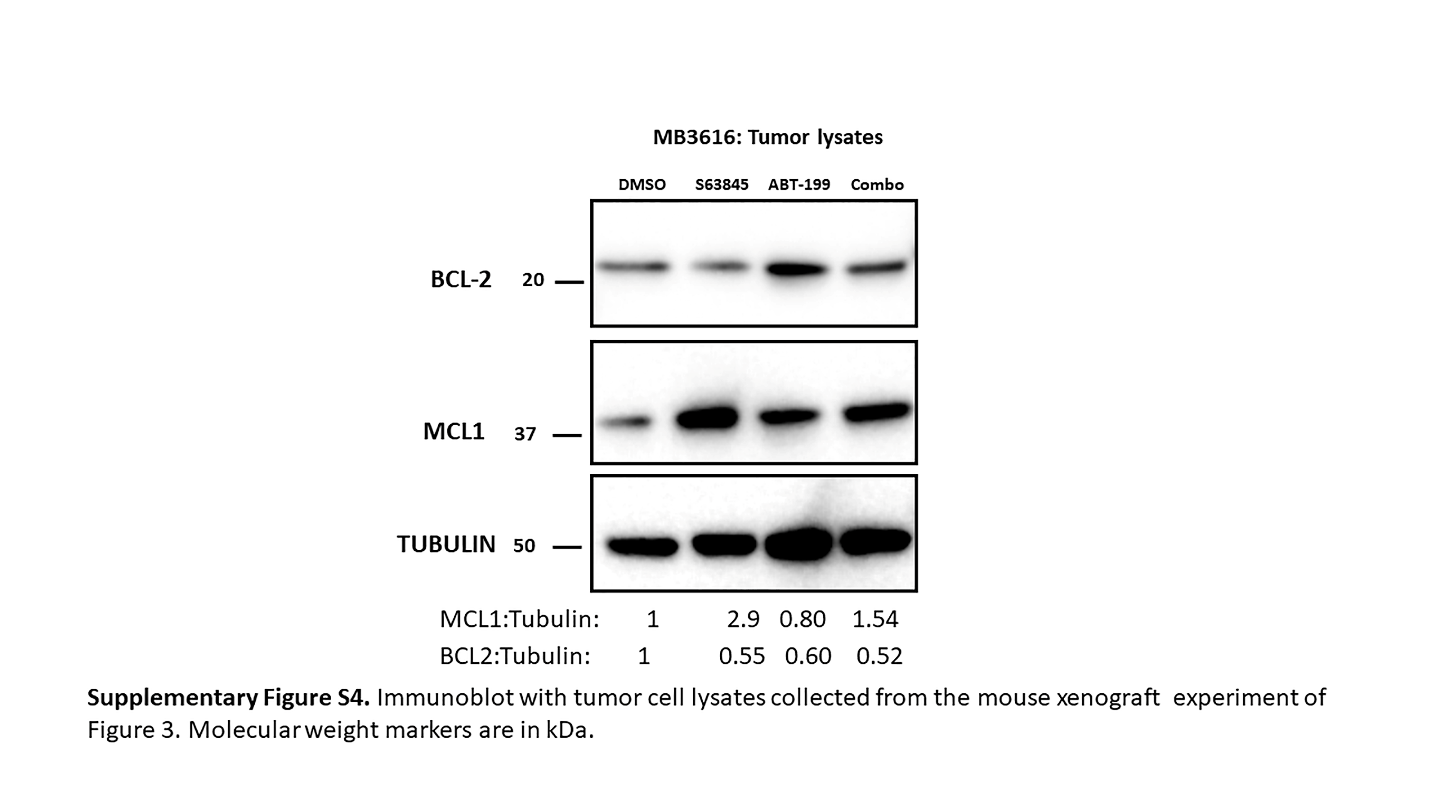


**Figure S4.** Immunoblot with tumor cell lysates collected from the mouse xenograft experiment of Figure 3. Molecular weight markers are in kDa.


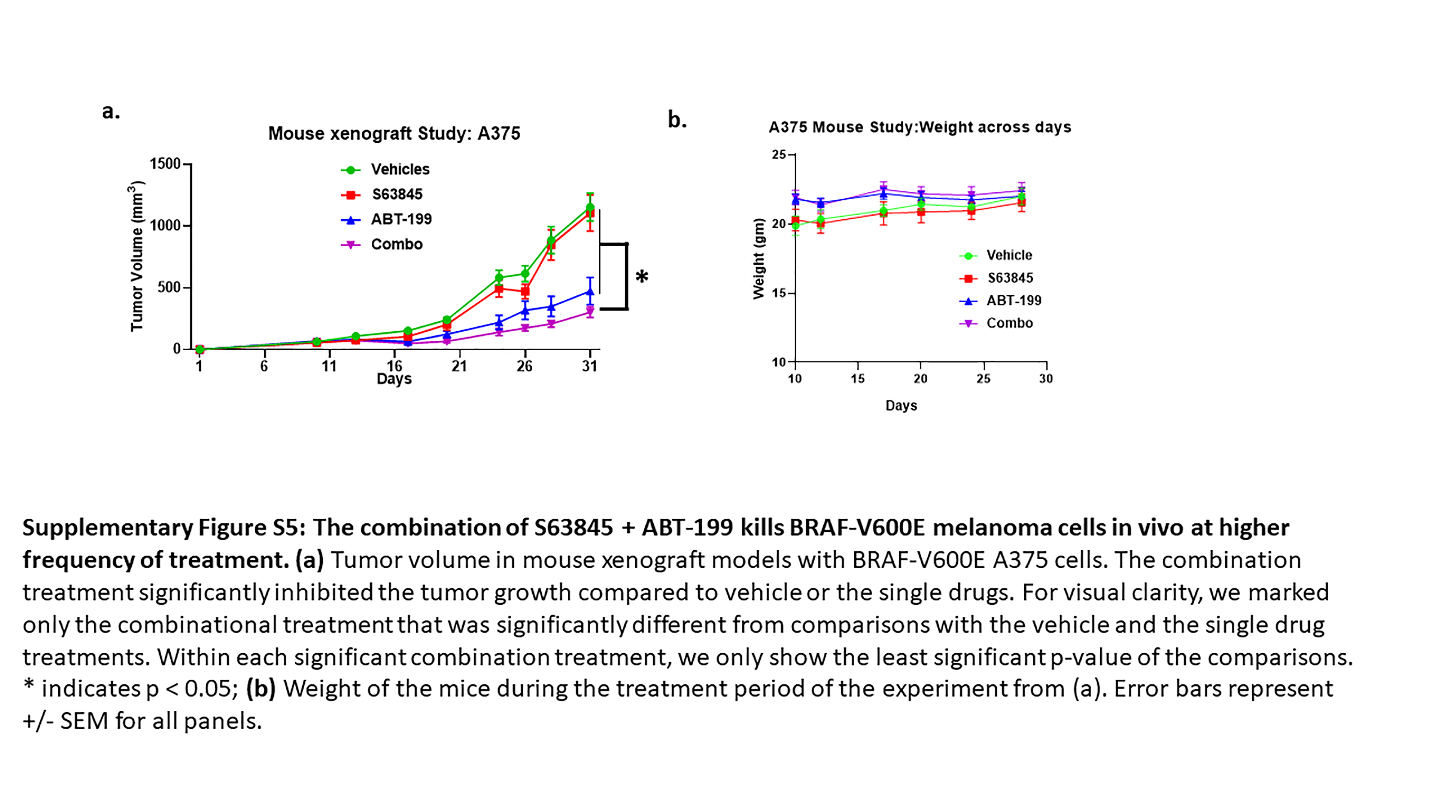


**Figure S5:** The combination of S63845 + ABT-199 kills BRAF-V600E melanoma cells in vivo at higher frequency of treatment. (**a**) Tumor volume in mouse xenograft models with BRAF-V600E A375 cells. The combination treatment significantly inhibited the tumor growth compared to vehicle or the single drugs. For visual clarity, we marked only the combinational treatment that was significantly different from comparisons with the vehicle and the single drug treatments. Within each significant combination treatment, we only show the least significant *p*-value of the comparisons. * indicates *p* < 0.05; (**b**) Weight of the mice during the treatment period of the experiment from (a). Error bars represent +/- SEM for all panels.


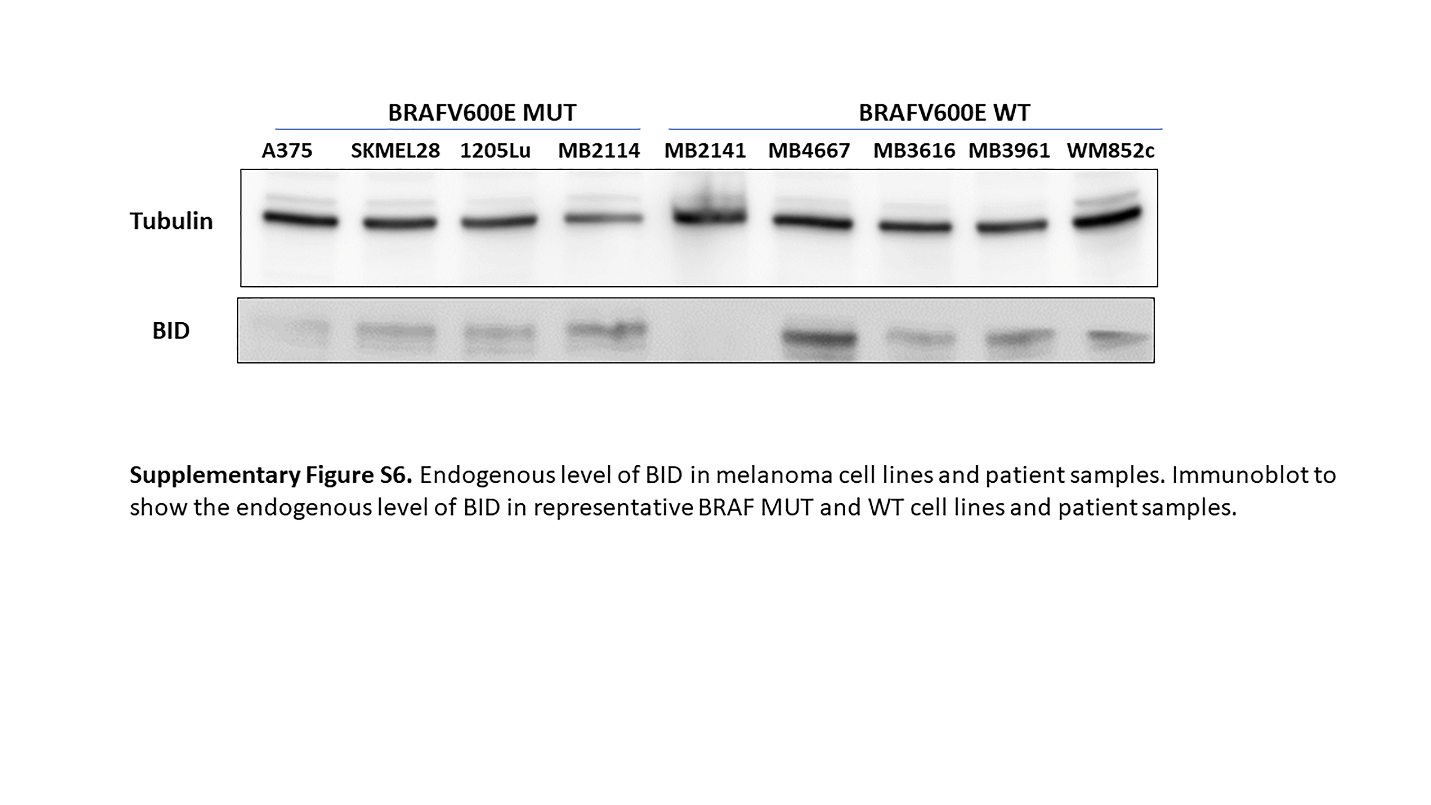


**Figure S6.** Endogenous level of BID in melanoma cell lines and patient samples. Immunoblot to show the endogenous level of BID in representative BRAF MUT and WT cell lines and patient samples.


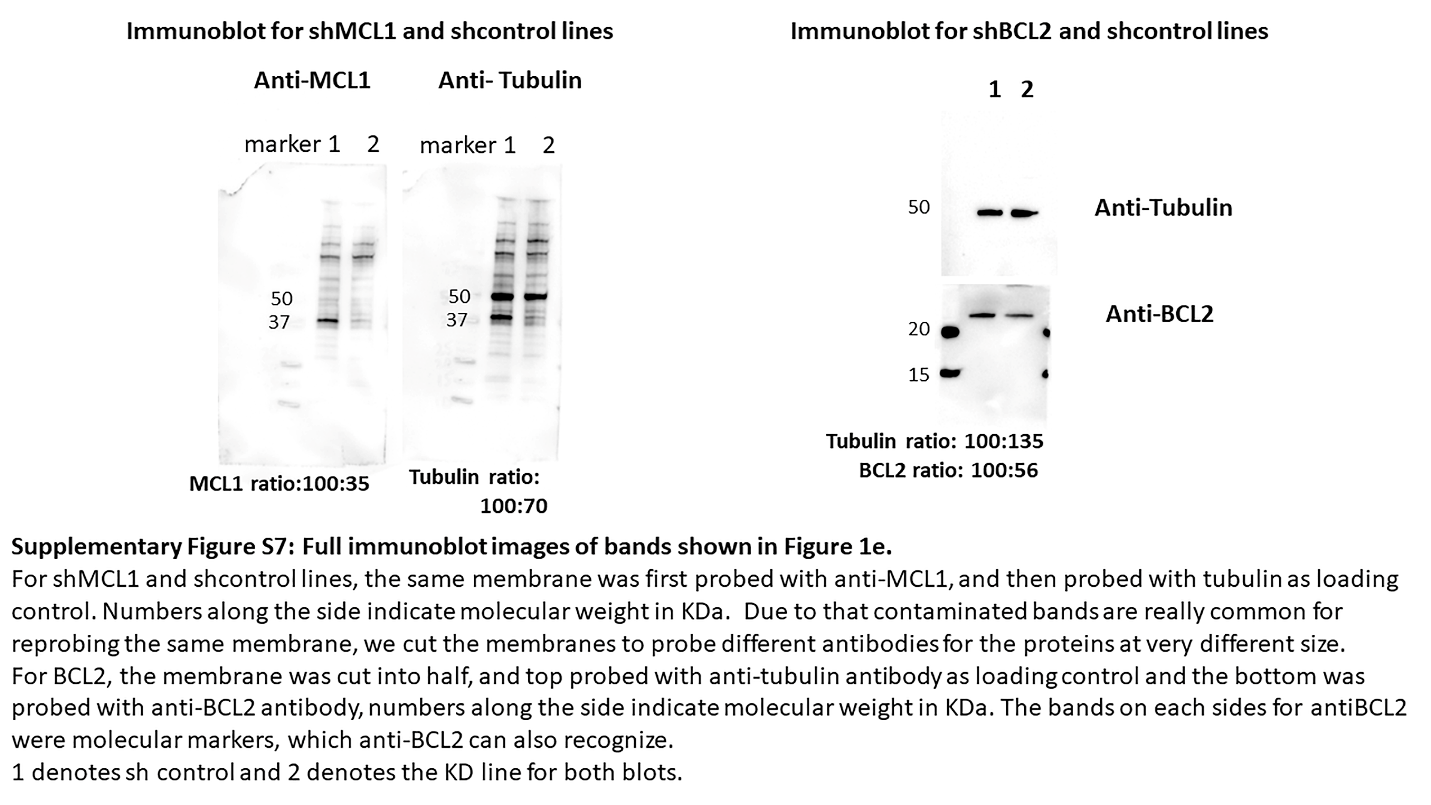


**Figure S7:** Full immunoblot images of bands shown in Figure 1e. For shMCL1 and shcontrol lines, the same membrane was first probed with anti-MCL1, and then probed with tubulin as loading control. Numbers along the side indicate molecular weight in KDa. Due to that contaminated bands are really common for reprobing the same membrane, we cut the membranes to probe different antibodies for the proteins at very different size. For BCL2, the membrane was cut into half, and top probed with anti-tubulin antibody as loading control and the bottom was probed with anti-BCL2 antibody, numbers along the side indicate molecular weight in KDa. The bands on each sides for antiBCL2 were molecular markers, which anti-BCL2 can also recognize. 1 denotes sh control and 2 denotes the KD line for both blots.


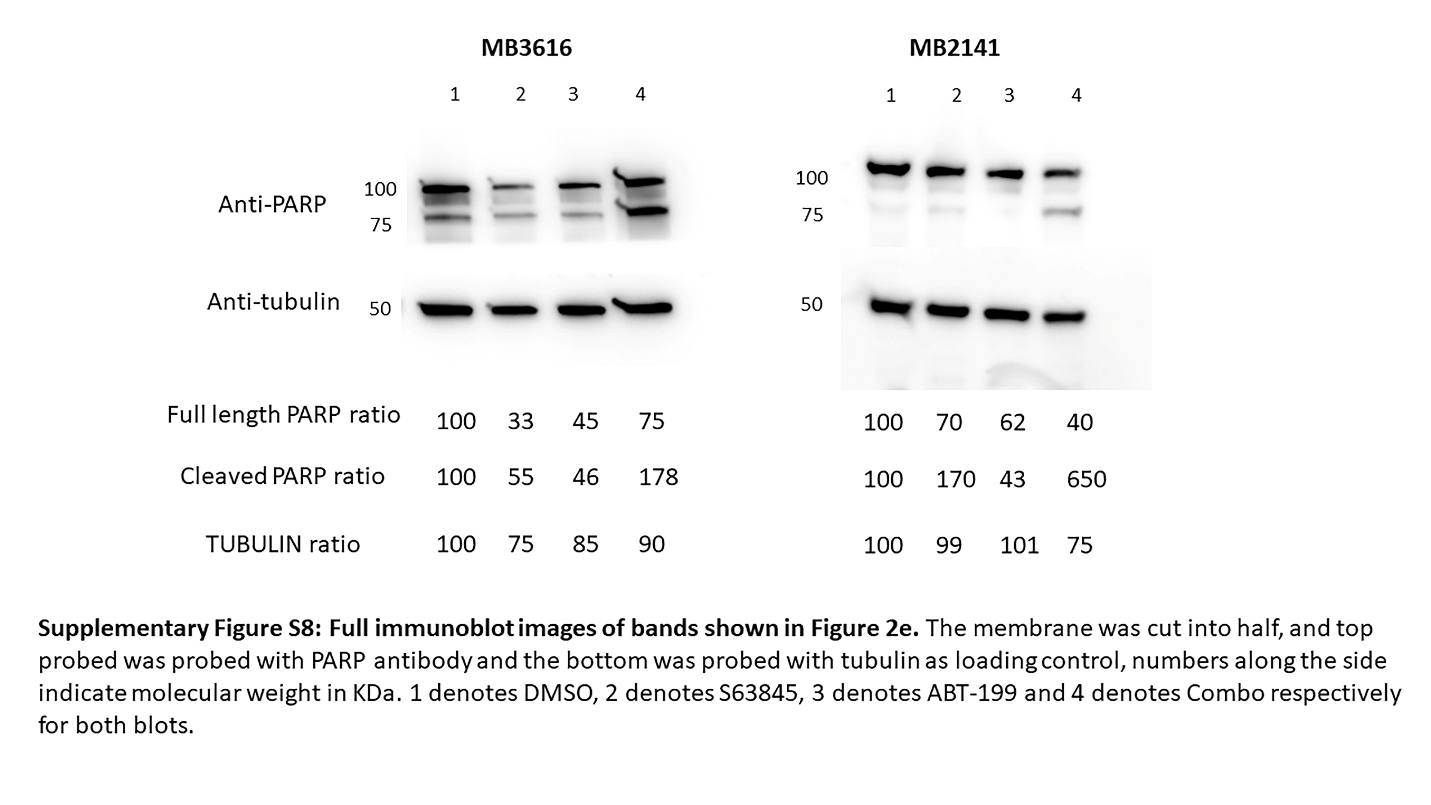


**Figure S8:** Full immunoblot images of bands shown in Figure 2e. The membrane was cut into half, and top probed was probed with PARP antibody and the bottom was probed with tubulin as loading control, numbers along the side indicate molecular weight in KDa. 1 denotes DMSO, 2 denotes S63845, 3 denotes ABT-199 and 4 denotes Combo respectively for both blots.


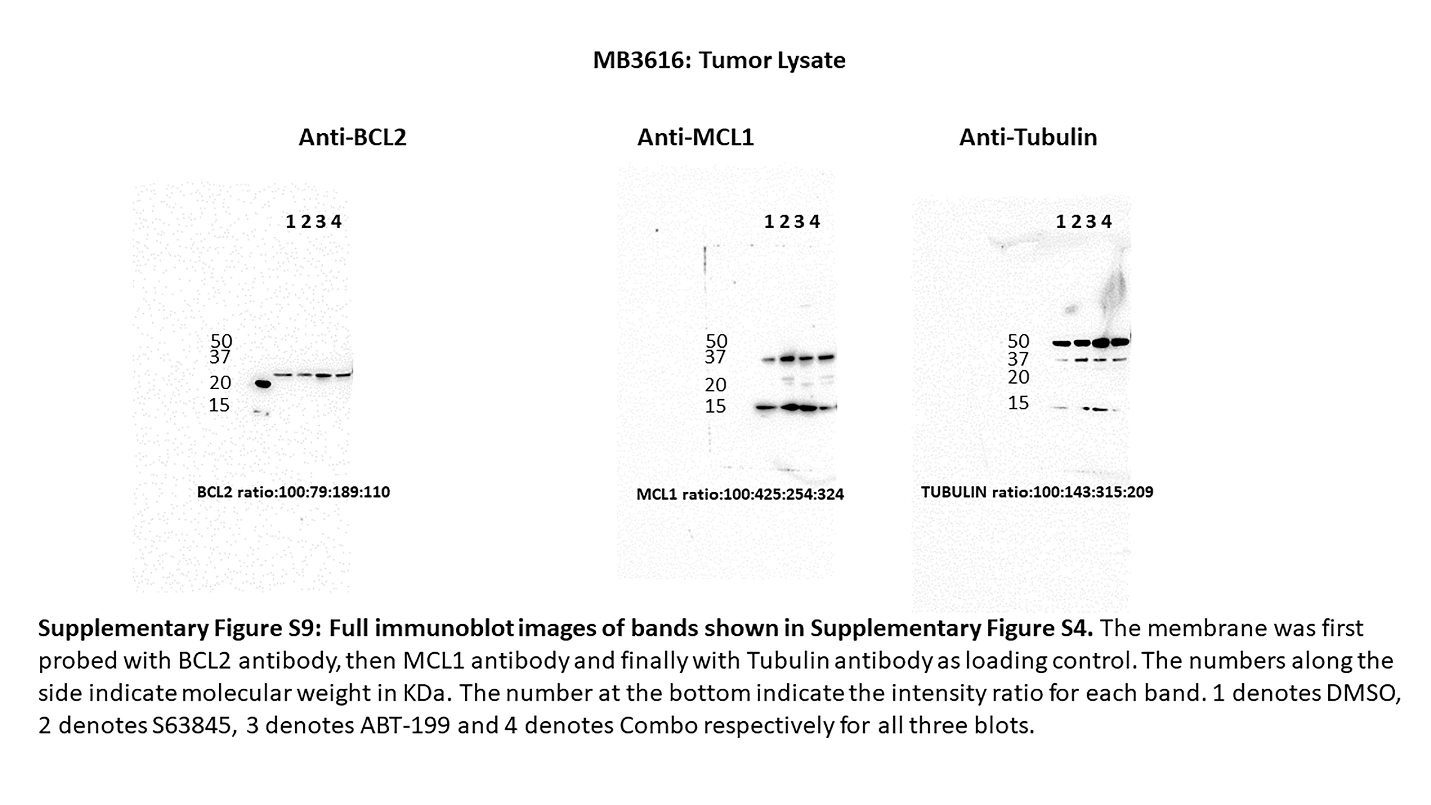


**Figure S9:** Full immunoblot images of bands shown in Supplementary Figure S4. The membrane was first probed with BCL2 antibody, then MCL1 antibody and finally with Tubulin antibody as loading control. The numbers along the side indicate molecular weight in KDa. The number at the bottom indicate the intensity ratio for each band. 1 denotes DMSO, 2 denotes S63845, 3 denotes ABT-199 and 4 denotes Combo respectively for all three blots.


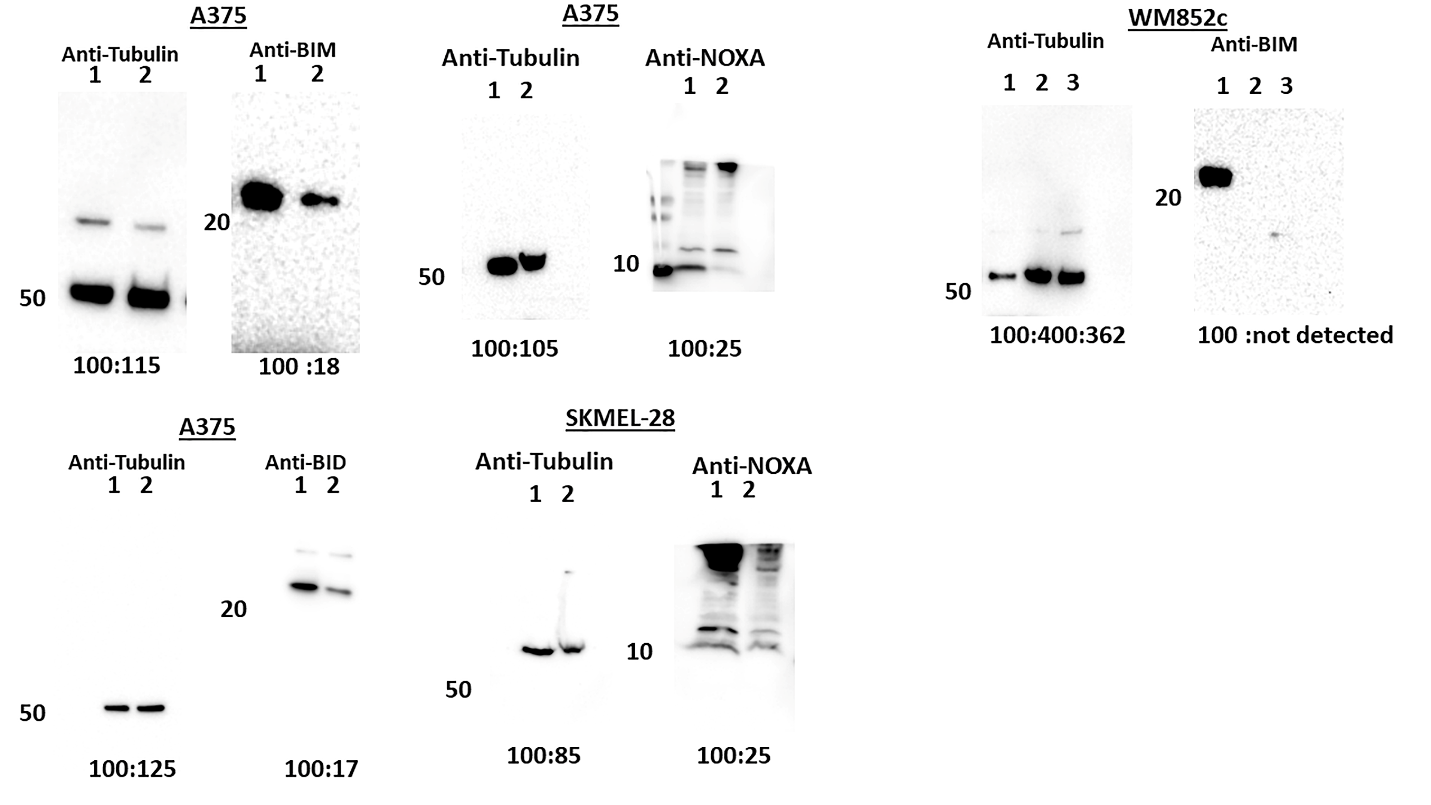


**Figure S10:** Full immunoblot images of bands shown in Figure 5. The membranes were cut into half, and top was probed with tubulin antibody as loading control and the bottom was probed with indicated protein. Numbers along the side indicate molecular weight in KDa. The numbers at the bottom indicate the intensity ratio for each band. 1 denotes sh control and 2 (and 3) denotes KD/KO line for all blots.


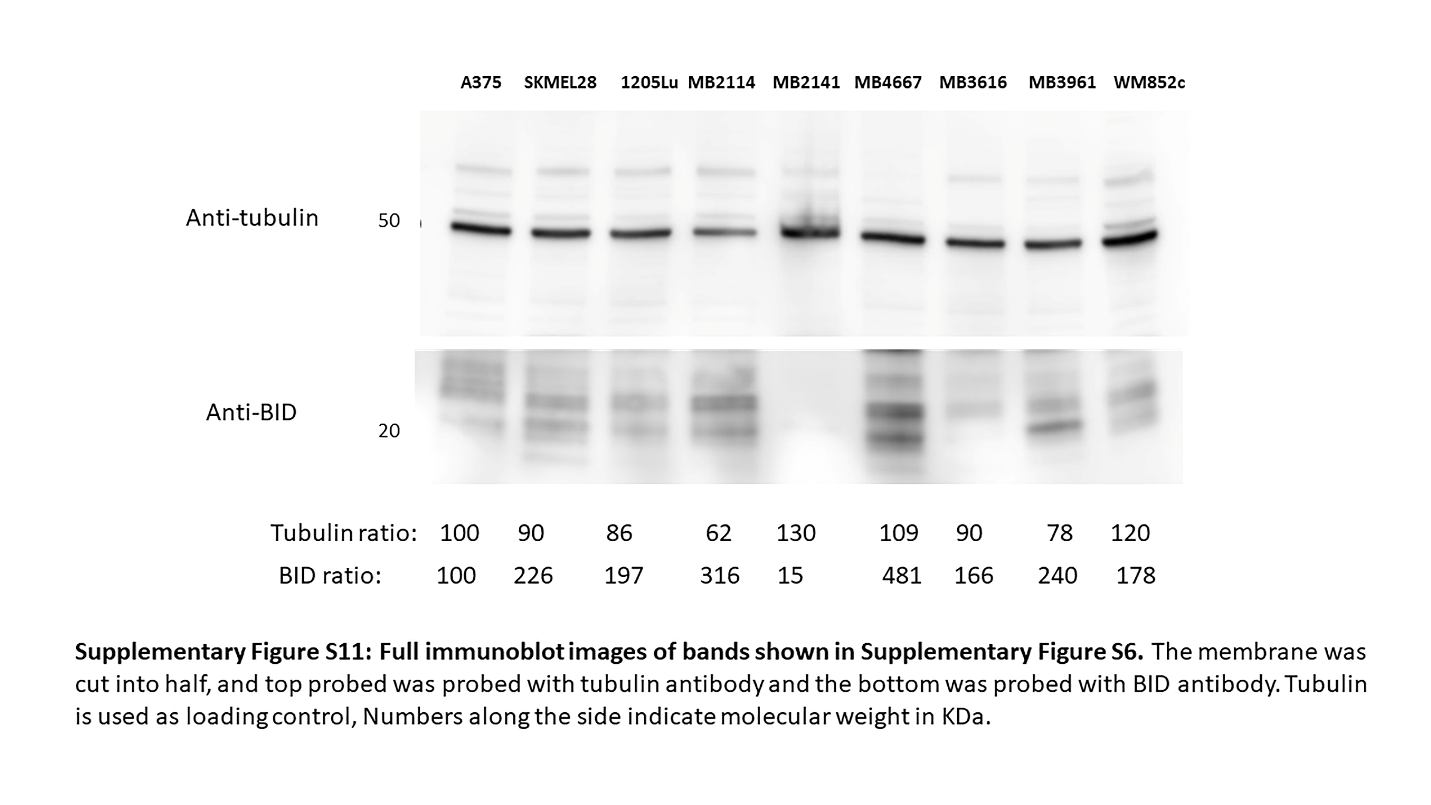


**Figure S11:** Full immunoblot images of bands shown in Supplementary Figure S6. The membrane was cut into half, and top probed was probed with tubulin antibody and the bottom was probed with BID antibody. Tubulin is used as loading control, Numbers along the side indicate molecular weight in KDa.

Supplementary Tables

**Table S3.** Details of the melanoma lines used in the study and IC50 of indicated drugs.

| **Cell/MB Line** | **Genotype** | **IC50 S63845 (μM)** | **IC50 ABT-199 (μM)** | **IC50**  **S63845+ABT-199 (μM)** | **Subtype** | **Response status to current standard of care** |
| --- | --- | --- | --- | --- | --- | --- |
| MB2114 | BRAF V600E | >10 | >10 | 1.886 | Unknown primary | BRAFi resistant |
| A375 | BRAFV600E | 6.335 | >10 | 1.871 | Cutaneous | N/A |
| 1205Lu | BRAFV600E | 7.195 | >10 | 1.414 | Cutaneous-Metastatic | N/A |
| SKMEL-28 | BRAFV600E | 4 | >10 | 1.271 | Cutaneous | N/A |
| MB2724 | Triple WT (WT for BRAF, NRAS and NF-1) | 3.242 | >10 | 0.6864 | Acral | N/A |
| MB1692 | AGK-BRAF | 7.415 | >10 | 0.637 | Superficial spreading | PD1 responder |
| MB2141 | EML4-ALK | 2.67 | >10 | 0.6286 | Mucosal | CTLA4 responder, treatment stopped and then had a relapse |
| Hs852T | WT for BRAF | 6.143 | 8.805 | 0.5368 | Cutaneous | N/A |
| MB3429 | BRAF G596C, GNA11 R183C | 8.854 | 3.91 | 0.5368 | Nodular | PD1 partial response |
| MB4667 | NRAS Q61R | >10 | 7.69 | 0.527 | Acral | Immunotherapy relapsed |
| Hs294T | WT for BRAF/NRAS | 2.901 | >10 | 0.485 | Cutaneous | N/A |
| MB3616 | NRAS Q61K | 8.107 | 9.69 | 0.452 | Superficial spreading | N/A |
| MB3961 | NRAS Q61K | 2.907 | >10 | 0.4539 | Nodular | Immunotherapy relapsed |
| MB3447 | Triple WT (WT for BRAF, NRAS and NF-1) | 5.49 | >10 | 0.348 | Superficial spreading | N/A |
| WM852c | NRAS | 7.811 | >10 | 0.145 | Cutaneous | N/A |

**Table S4.** *p* values for ATP assay of S63845+ABT-199 Combination (Figure 2a).

Dose of 156nM

|  | **DMSO vs Combo** | **ABT-199 vs Combo** | **S63845 vs Combo** |
| --- | --- | --- | --- |
| MB3447 | <0.0001 | <0.0001 | <0.0001 |
| MB3429 | <0.0001 | <0.0001 | <0.0001 |
| MB4667 | <0.0001 | 0.0002 | <0.0001 |
| MB2114 | ns | ns | ns |

Dose of 625nM

|  | **DMSO vs Combo** | **ABT-199 vs Combo** | **S63845 vs Combo** |
| --- | --- | --- | --- |
| MB3447 | <0.0001 | <0.0001 | <0.0001 |
| MB3429 | <0.0001 | <0.0001 | <0.0001 |
| MB4667 | <0.0001 | <0.0001 | <0.0001 |
| MB2114 | ns | ns | ns |

Dose of 2.5µM

|  | **DMSO vs Combo** | **ABT-199 vs Combo** | **S63845 vs Combo** |
| --- | --- | --- | --- |
| MB3447 | <0.0001 | <0.0001 | <0.0001 |
| MB3429 | <0.0001 | <0.0001 | <0.0001 |
| MB4667 | <0.0001 | <0.0001 | <0.0001 |
| MB2114 | <0.0001 | <0.0001 | <0.0001 |

Dose of 10µM

|  | **DMSO vs Combo** | **ABT-199 vs Combo** | **S63845 vs Combo** |
| --- | --- | --- | --- |
| MB3447 | <0.0001 | <0.0001 | <0.0001 |
| MB3429 | <0.0001 | <0.0001 | <0.0001 |
| MB4667 | <0.0001 | <0.0001 | <0.0001 |
| MB2114 | <0.0001 | <0.0001 | <0.0001 |

The numbers indicate the p values. ns denotes not significant.

**Table S5.** *p* values for Figure 4.

P values for Primary Sphere Assay (Figure 4a)

|  | **DMSO vs Combo** | **ABT-199 vs Combo** | **S64315 vs Combo** |
| --- | --- | --- | --- |
| A375 | 0.0061 | 0.0025 | 0.0053 |
| 1205Lu | 0.0016 | 0.0006 | 0.0019 |
| SKMEL-28 | 0.0036 | 0.0016 | 0.0029 |
| MB3429 | 0.0253 | 0.0130 | 0.0069 |
| MB4667 | <0.0001 | <0.0001 | 0.0021 |
| MB3616 | <0.0001 | 0.0004 | <0.0001 |
| MB2141 | 0.0001 | 0.0001 | 0.0010 |
| WM852c | 0.0036 | 0.0016 | 0.0029 |

P values for Secondary Sphere Assay (Figure 4d)

|  | **DMSO vs Combo** | **ABT-199 vs Combo** | **S64315 vs Combo** |
| --- | --- | --- | --- |
| A375 | 0.0002 | 0.0057 | <0.0001 |
| 1205Lu | 0.0030 | 0.0030 | 0.0035 |
| MB3616 | 0.0030 | 0.0059 | 0.0277 |
| MB4667 | <0.0001 | <0.0001 | 0.0009 |
| WM852c | <0.0001 | <0.0001 | <0.0001 |

The numbers indicate the p values.

**Table S6.** *p* values for Figure 6.

Dose of 156nM

|  | **DMSO vs Combo** | **ABT-199 vs Combo** | **S64315 vs Combo** |
| --- | --- | --- | --- |
| MB2141 | <0.0001 | <0.0001 | <0.0001 |
| MB3616 | <0.0001 | <0.0001 | <0.0001 |
| MB3961 | <0.0001 | <0.0001 | <0.0001 |
| MB4667 | <0.0001 | <0.0001 | <0.0001 |
| A375 | ns | ns | ns |
| 1205Lu | ns | 0.0138 | ns |

Dose of 625nM

|  | **DMSO vs Combo** | **ABT-199 vs Combo** | **S64315 vs Combo** |
| --- | --- | --- | --- |
| MB2141 | <0.0001 | <0.0001 | <0.0001 |
| MB3616 | <0.0001 | <0.0001 | <0.0001 |
| MB3961 | <0.0001 | <0.0001 | <0.0001 |
| MB4667 | <0.0001 | <0.0001 | <0.0001 |
| A375 | <0.0001 | <0.0001 | <0.0001 |
| 1205Lu | <0.0001 | <0.0001 | <0.0001 |

Dose of 2.5µM

|  | **DMSO vs Combo** | **ABT-199 vs Combo** | **S64315 vs Combo** |
| --- | --- | --- | --- |
| MB2141 | <0.0001 | <0.0001 | <0.0001 |
| MB3616 | <0.0001 | <0.0001 | <0.0001 |
| MB3961 | <0.0001 | <0.0001 | <0.0001 |
| MB4667 | <0.0001 | <0.0001 | <0.0001 |
| A375 | <0.0001 | <0.0001 | <0.0001 |
| 1205Lu | <0.0001 | <0.0001 | <0.0001 |

Dose of 10µM

|  | **DMSO vs Combo** | **ABT-199 vs Combo** | **S64315 vs Combo** |
| --- | --- | --- | --- |
| MB2141 | <0.0001 | <0.0001 | <0.0001 |
| MB3616 | <0.0001 | <0.0001 | <0.0001 |
| MB3961 | <0.0001 | <0.0001 | 0.0196 |
| MB4667 | <0.0001 | <0.0001 | <0.0001 |
| A375 | <0.0001 | <0.0001 | <0.0001 |
| 1205Lu | <0.0001 | <0.0001 | ns |

The numbers indicate the p values. ns denotes not significant.
